# Hope for Ostomates: A Carbon and Zeolite Impregnated Polyester Fabric Inhibits Urine Odor in Cancer Patients: A Randomized Experimental Study

**DOI:** 10.31557/APJCP.2021.22.9.2917

**Published:** 2021-09

**Authors:** Gianluigi Taverna, Linda M. Thiel, Desiree L. Miller, Lorenzo Tidu, Paolo Sardella, Patricia Camp, Matteo Luigi Zanoni, Paolo Vota, Cinzia Mazzieri, Giovanni Toia, Vittorio Fasulo, Pierpaolo Avolio, Alessio Benetti, Niccolò Buffi, Giovanni Lughezzani, Massimo Lazzeri, Paolo Casale, Giorgio Guazzoni, Fabio Grizzi, Brian Stork

**Affiliations:** 1 *Department of Urology, Humanitas Mater Domini, Castellanza, Varese, Italy. *; 2 *Department of Urology, IRCCS Humanitas Research Hospital, Rozzano, Milan, Italy. *; 3 *McAuley School of Nursing, College of Health Professions, University of Detroit Mercy, Detroit, Michigan, USA. *; 4 *Mercy Health Visiting Nurses Services and Hospice Services, Muskegon, Michigan, USA. *; 5 *Italian Ministry of Defenses, Military Veterinary Center, CEMIVET, Grosseto, Italy. *; 6 *Muskegon Ostomy Association, Muskegon, Michigan, USA. *; 7 *Department of Biomedical Sciences, Humanitas University, Pieve Emanuele, Milan, Italy. *; 8 *Department of Immunology and Inflammation, IRCCS Humanitas Research Hospital, Rozzano, Milan, Italy. *; 9 *University of Michigan, Department of Urology, Ann Arbor, Michigan, USA. *

**Keywords:** Bladder cancer, prostate cancer, stoma, ostomy pouch, urine odor

## Abstract

Objective:

Many individuals with bladder cancer have undergone a surgical urostomy and often complain of being self-conscious of the unpleasant smell of their own urine. The focus of this study was to test the efficacy of a pouch cover made of a carbon and zeolite containing polyester material to inhibit the smell of urine by comparing two trained dogs’ response time in detecting volatile organic compounds (VOCs) in urine, with and without the fabric covering the samples.

Methods:

This study used a randomized, blinded experimental design to evaluate the efficacy of a fabric to interfere with two highly trained dogs’ ability to detect specific VOCs present in the urine of prostate cancer patient. Ninety urine samples were analyzed in this study.

Results:

Prior to the experiment, both dogs accurately detected VOCs in the uncovered test urine samples of men with prostate cancer with a sensitivity and specificity of nearly 100%. Both dogs recognized the “uncovered” urine samples of men with prostate cancer within two seconds. When the test sample was covered with the study fabric, the test urine samples were detected within 30-40 seconds and in some instances the dogs were not able to identify the covered samples, whatsoever.

Conclusion:

The findings of this study demonstrate that the carbon and zeolite containing polyester fabric did significantly interfere with the ability of the dogs to detect VOCs in urine of men with prostate cancer. The fabric may show promise as a pouch cover in controlling offensive urine odor which many ostomates experience.

## Introduction

Several patients with advanced bladder cancer, which is ranked ninth worldwide and is the fifth most common type of cancer in Europe (Siegel et al., 2018; Mossanen, 2021; Siegel et al., 2021), undergo a surgical ostomy i.e. urostomy at the time of cystectomy or bladder removal (Goltz et al., 2021; Wulff-Burchfield et al., 2021). Patients who have undergone a surgical urostomy often complain of being self-conscious of the smell of their own urine. Additionally, many of these individuals ultimately undergo chemotherapy, which can result in a common “side-effect” of increased sensitivity to smell often conveyed as unpleasant and may negatively affect quality of life (Zabernigg et al., 2010). Bernhardson et al., (2009) reported this “side-effect” as a common concern among patients receiving chemotherapy. For nearly two decades, numerous studies have demonstrated the adverse effects of ostomy odor and appliance leakage on ostomates’ quality of life (Gooszen et al., 2000; Mitchell et al., 2007; Grant et al., 2011; Furukawa et al., 2013; Ratliff, 2014). Mitchell et al., (2007) reported a high level of embarrassment associated with leakage from appliances, gas, odors and noise from ostomies (ileostomy, colostomy) among Veterans. Furthermore, anxiety and depression were associated with embarrassment. Ratliff found similar concerns of leakage, odor, and gas among ostomates (Ratliff, 2014). Other findings suggest physical issues such as gas and odor are not dissimilar between gender (Grant et al., 2011) or type of ostomy (Gooszen et al., 2000; Furukawa et al., 2013). The ostomy literature is clear concerning the impact of odor on the ostomate’s quality of life. It is therefore apparent that controlling pouch odor can be a tenacious ordeal for ostomates. There are various commercial products available to control pouch odor, such as liquid deodorizers, charcoal pouch filters, deodorizer pads, and pouch covers. In 2015, an online community member recommended using breath mints to control pouch bag odor. Studies addressing ostomy pouch covers for control of odor are limited, if non-existent. A Pubmed and Cumulative Index to Nursing and Allied Health Literature (CINAHL) literature search by the authors did not uncover any research articles addressing fabric ostomy pouch covers for odor control. The focus of this study was to test the efficacy of a pouch cover made of a carbon and zeolite containing polyester material to inhibit the smell of urine (StomaCloak^®^, http://www.stomacloak.com). The ability of humans to perceive odor or smells varies widely (Jiang and Matsunami, 2015; Silva Teixeira et al., 2016). This makes it difficult to objectively evaluate. It has been shown that vertebrates, including canines and rats, and invertebrates, such as wasps, honeybees and moths, can be trained to identify and discriminate specific volatile odors (Schmidt, 2009; Leitch et al., 2013; Fischer-Tenhagen et al., 2017; Panebianco et al., 2017; Fischer-Tenhagen et al., 2018; Oh et al., 2021). Weetjens et al., (2009) reported that African giant-pouched rats (Crietomys gambianus) were successfully trained to identify Mycobacterium tuberculosis in sputa of patients suspected of having tuberculosis, with a sensitivity of 89.1% and specificity of 80.1%. The use of sniffer dogs has a long history and a wider variety of applications, including the detection of explosives or land mines, the detection of drugs or the search of missing peoples, health-threatening microbial contaminations in hospitals and apartments (Fischer-Tenhagen et al., 2013; Fischer-Tenhagen et al., 2017; Else, 2020; Grandjean et al., 2020; Mazzola et al., 2020; Guest et al., 2021). Dogs are thought to poses an extremely keen sense of smell that is reported to be 10000 to 100000 times more accurate than in humans (Rygg et al., 2017; Else, 2020). Some studies have reported thresholds ranging from parts-per-million to part-for-trillion (Leitch et al., 2013). It has also been reported that highly trained dogs can accurately detect distinctive odors, also named volatile organic compounds (VOCs) that are present in urine of men with prostate cancer as products of cell metabolism (Willis et al., 2004; Gordon et al., 2008; Cornu et al., 2011; Jobu et al., 2012; Leitch et al., 2013; Taverna et al., 2015; Taverna et al., 2016; Guest et al., 2021). As dogs can reliably detect these compounds, it follows that they could also be used to test scent control products, such as a fabric stoma pouch cover. The literature also reports the reliability of dogs to detect distinctive VOC urine odors (Taverna et al., 2015; Taverna et al., 2016; Guest et al., 2021). In this study, we aimed to evaluate the efficacy of a fabric to reduce or inhibit urine odor. The material is a carbon and zeolite i.e. microporous, aluminosilicate mineral to “adsorb’ odor molecules” impregnated polyester fabric. The study compared two trained dogs’ response time in detecting the scent of urine, with and without the fabric covering the urine samples. This study sought to answer the following research question: What is the effect of a carbon and zeolite impregnated polyester fabric on the ability of dogs to sniff VOCs in urine of men with prostate cancer as compared to urine of men without prostate cancer?

## Materials and Methods

The study was approved by the Ethics Committee at Humanitas Clinical and Research Center, Rozzano, Milan, Italy where patients were treated (CE-ICH260/11). Each participant was informed about the study and provided informed consent as participants’ urine was used for the prostate cancer VOC test and control samples. This study used a randomized, blinded experimental design to evaluate the efficacy of the fabric to interfere with the ability of highly trained dogs to detect prostate cancer-specific VOCs. The “test” samples were urine with VOCs of men with prostate cancer, while the “control” samples were prostate cancer free urine. The test sample was randomly assigned prior to each dog run. Dog handlers were blinded from test- and control urine samples. Samples of the carbon and zeolyte containing polyester fabric were sent to the study’s researchers (G.T & F.G) at the Humanitas Research Hospital (Rozzano, Milan, Italy) by one of the study’s authors and product’s sponsors (B.S). The fabric, along with samples of control and prostate cancer patient urine were then transported to the Italian Ministry of Defense’s, Military Veterinary Center, CEMIVET, Grosseto, Italy. A military Chief medial veterinary surgeon (L.T) familiar with the protocol supervised the study. The center also houses two German Shepard Explosive Detection dogs and their handlers. In addition to being trained to detect explosives, the dogs have been trained to detect the VOCs in urine samples of men with prostate cancer with high sensitivity and specificity (Taverna et al., 2015; Taverna et al., 2016).

Ninety urine samples were analyzed in this study. Among these, 15 urine samples were collected from men with prostate cancer at various grades and stages (4 patients with Gleason score 3+3; 6 patients with Gleason score 4+3; 3 patients with Gleason score 4+4; 3 patient and 2 patients with metastatic prostate cancer, of which one under hormonal therapy and one without). The control group consisted of 75 urine samples collected from men without prostate cancer (10 patients with bladder cancer, 4 with kidney cancer, 1 with pancreatic cancer, 2 with breast cancer, 3 with diabetes mellitus type I, 4 with hyperthyroidism, 5 with prostatitis and 46 healthy volunteer donors). Each run (each dog sniffing 6 urine samples) included 1 sample collected from men with prostate cancer and 5 samples randomly selected from the control group. The positioning of the positive samples was always randomly determined by ad hoc software. The dogs reported the positive sample only after making the full run twice. Both dogs had been trained to sit when they detected VOCs in the urine samples of men with prostate cancer and were rewarded with praise and play when they identified the sample with prostate cancer VOCs correctly. Multiple sample runs were made to verify that both dogs could accurately detected VOCs from the test-scent urine sample “without” the fabric covering the urine sample. The run was then repeated; however, this time the test fabric was placed securely over the test-scent urine sample from the prostate cancer patients. During this phase both dogs and their respective handlers were blinded to the identity of the samples. In addition, all of the samples were presented such that there were no visual hints as to which sample had been covered with the study material. Data were expressed as mean values ± SD, and were analyzed using GraphPad Prism 5 (San Diego, California, USA). Univariate analysis was performed by means of the two-tailed Student’s test for parametric variables. A P-value of ≤0.05 was considered to be statistically significant.

## Results

Prior to the experiment, both dogs accurately detected VOC’s in the uncovered test urine samples of men with prostate cancer with a sensitivity and specificity of 100%. The dogs accurately reported the uncovered test after making the full run twice. Both dogs recognized the uncovered samples within 2 seconds (mean: 1±0,5 seconds; range: 1-2 seconds). When the test sample was covered with the study fabric, Dog 1 reported the test sample after making the full run twice, recognizing the covered urine samples of men with prostate cancer after a mean of 35±3 seconds (range: 30-40 seconds) in 14 out of 15 attempts. The 15th sample (i.e. metastatic prostate cancer under hormonal therapy) was not recognized after 60 seconds and the dog was discharged from the setting room. Dog 2 reported the test sample after making the full run twice, recognizing the covered urine samples of men with prostate cancer after a mean period of 35±4 seconds (range: 30-40 seconds) in 13 out of 15 attempts. The two cases (urine of men with prostate cancer, case 14 and 15) were not recognized after 60 seconds and the dog was discharged from the setting room. Overall, in 3 out of 30 cases (10%), the fabric inhibited the dogs’ ability to detect VOCs in prostate urine, “all together”. The fabric significantly (p = <0.0001) inhibited the ability of the dogs to detect urine of men with prostate cancer-specific VOCs as compared to urine samples not covered by the material.

## Discussion

Patient with a surgical urostomy can experience a variety of quality of life (QOL) challenges. Odor from the smell of urine can negative impact QOL in patients with ostomies. There are a number of products available to ostomates that reportedly can assist in controlling the odor of urine from an ostomy device, however scientific data as to product efficacy is rare. Data obtained from this study demonstrates that a proprietary carbon and zeolyte impregnated polyester material significantly interferes with the dog’s ability to sniff VOCs present in urine samples of men with prostate cancer. There were times (1 out of 15 samples and 2 out of 15 samples) when the fabric prevented the dogs from detecting the prostate cancer VOCs all together (not detected after 60 seconds). The dogs in this study were chosen because of their accuracy in detecting prostate cancer VOCs in human urine; a strength of the study. The randomized, blinded experimental design is another strength of this study, as it controls for confounding variables. Further studies are needed to determine if this material can be used to block VOCs in human stool. If the material can perform in this fashion, it could potentially be used to benefit patients with fecal incontinence (i.e. colostomy and ileostomy). 

Dating back to 400 BC, Hippocrates recognized the diagnostic value of odors, but the industrial and technological revolution of the 18th and 19th centuries changed the history of medical diagnosis, and odors were neglected in this period (Pavlou and Turner, 2000; Fischer-Tenhagen et al., 2013). Hundreds of volatile organic compounds (VOCs) are emitted from the human body, and the components of VOCs usually reflect the metabolic condition of an individual (Shirasu and Touhara, 2011). All organisms are required to make appropriate decisions depending on their situation in their environment in order to maintain their life and conserve their species. Chemical communication is widely used among various organisms. Although a large variety of molecules have been shown to act as chemical cues, the molecular and neural basis underlying the behaviors elicited by these molecules has been revealed for a small number of molecules. It is also known that olfaction is essential for the survival of most mammals (Niimura et al., 2014). In general terms, olfactory acuity is thought to be attributable to olfactory organ size (e.g., size of the olfactory bulb, surface area of olfactory epithelium), the density of olfactory sensory neurons in the olfactory epithelium, and the number and diversity of functional genes versus pseudogenes in the olfactory receptor gene family. Additionally, nasal morphology is thought to play a critical role in olfaction, as it controls airflow and odorant deposition in the nose. Specifically, the gross anatomical structure of the nasal cavity and the configuration of the olfactory region (particularly the presence or absence of an “olfactory recess”) control the transport of odorant-laden air from the external environment to the sensory part of the nose, which is the first critical step in olfaction (Kaupp, 2010; Jiang and Matsunami, 2015; Wackermannova et al., 2016).

Both dogs were not trained simply to detect the presence or absence of urine. Rather, these dogs were specifically trained to detect prostate cancer VOCs in human urine (Taverna et al., 2015; Taverna et al., 2016). It is uncertain if the prostate VOCs the dogs sniffing are perceptible to humans and, therefore, are the cause of perceptible urine odor in humans. However, it is well known that, even in that absence of prostate cancer, urine has a distinctive, often unpleasant smell. Ostomy odor affects the QOL of ostomates (Vonk-Klaassen et al., 2016). The findings of this study can assist ostomates and nurses in making self-management decisions regarding odor associated with stoma pouches. We found that a polyester material impregnated with activated carbon and zeolyte can interfere with highly trained, military dogs from detecting the volatile organic compounds present in urine samples of men with prostate cancer. The use of the fabric delayed recognition from 1-2 seconds (no fabric) to 30-40 seconds (with fabric) and is considered a substantial clinical finding. In three cases the dogs were not able to detect the covered test urine “whatsoever”. It follows that data from this study can be extrapolated and transferred to non-pathologic human urine. Based on this study, the fabric shows promise in controlling urine odor. To our knowledge, this study is the first to evaluate a fabric’s ability to inhibit urine stoma pouch odor. In conclusion, we demonstrated that carbon and zeolite impregnated polyester fabric might inhibits urine odors. This finding stimulated new studies on the interactions between these chemical compounds and the urine specific VOCs. 

## Author Contribution Statement

The authors confirm contribution to the paper as follows: study conception and design: GT, BS, LT, FG; data collection: GT, BS, LT, PS, FG; analysis and interpretation of results: GT, BS, LT, FG; draft manuscript preparation: GT, BS, LT, LMT, DLM, FG, PC. All authors reviewed and discussed the results and approved the final version of the manuscript.
